# Adjuvant Chemoradiotherapy Associated with Improved Overall Survival in Resected Esophageal Squamous Cell Carcinoma after Neoadjuvant Chemoradiotherapy in Intensity-Modulated Radiotherapy Era

**DOI:** 10.3390/biomedicines10112989

**Published:** 2022-11-21

**Authors:** Wing-Keen Yap, Ming-Chieh Shih, Yu-Chen Chang, Chia-Hsin Lin, Shih-Ming Huang, Tsung-You Tsai, Ching-Fu Chang, Chih-Chung Hsu, Chen-Kan Tseng, Miao-Fen Chen, Din-Li Tsan, Chi-Ting Liau, Ming-Mo Hou, Yin-Kai Chao, Chien-Hung Chiu, Tsung-Min Hung

**Affiliations:** 1 Proton and Radiation Therapy Center, Chang Gung Memorial Hospital-Linkou Medical Center, Department of Radiation Oncology, Chang Gung University, 5 Fu-Shin Street, Kwei-Shan, Taoyuan 333, Taiwan; 2 Department of Applied Mathematics, National Dong Hwa University, Hualien 974, Taiwan; 3 Department of Radiation Oncology, Keelung Chang Gung Memorial Hospital, Keelung 204, Taiwan; 4 Chang Gung Memorial Hospital-Linkou Medical Center, Department of Otolaryngology-Head and Neck Surgery, Chang Gung University, 5 Fu-Shin Street, Kwei-Shan, Taoyuan 333, Taiwan; 5 Chang Gung Memorial Hospital-Linkou Medical Center, Department of Hematology-Oncology, Chang Gung University, 5 Fu-Shin Street, Kwei-Shan, Taoyuan 333, Taiwan; 6 Chang Gung Memorial Hospital-Linkou Medical Center, Department of Thoracic Surgery, Chang Gung University, 5 Fu-Shin Street, Kwei-Shan, Taoyuan 333, Taiwan

**Keywords:** neoadjuvant chemoradiotherapy, trimodality therapy, adjuvant chemoradiotherapy, IMRT, VMAT, esophageal cancer, squamous cell carcinoma, post-neoadjuvant pathologic stage, ypT3, ypN+

## Abstract

Background: The prognosis of patients with resected esophageal squamous cell carcinoma after neoadjuvant chemoradiotherapy is particularly poor in those who were staged as ypT3/T4 and/or ypN+. This study investigated whether adjuvant chemoradiotherapy was associated with improved clinical outcomes in these patients. Methods: we identified patients with esophageal squamous cell carcinoma who were staged as ypT3/T4 and/or ypN+ after being treated with neoadjuvant chemoradiotherapy followed by esophagectomy between the years 2013 and 2019. Patients were divided into two groups based on whether they received adjuvant chemoradiotherapy. The Kaplan-Meier method and Cox regression modeling were performed for survival analyses and multivariable analysis, respectively. Results: 76 eligible patients were included in the analyses. The median follow-up for the study cohort was 43.4 months. On Kaplan-Meier analyses of the overall population, adjuvant chemoradiotherapy was associated with significantly improved median overall survival (31.7 months vs. 16.3 months, *p* = 0.036). On Kaplan-Meier analyses of the 35 matched pairs generated by propensity score matching, adjuvant chemoradiotherapy was associated with significantly longer median overall survival (31.7 months vs. 14.3 months; *p* = 0.004) and median recurrence-free survival (18.9 months vs. 11.7 months; *p* = 0.020). In multivariable analysis, adjuvant chemoradiotherapy was independently associated with a 60% reduction in mortality (*p* = 0.003) and a 48% reduction in risk of recurrence (*p* = 0.035) after adjusting for putative confounders. In addition, microscopic positive resection margin and Mandard tumor regression grade 3–4 were independently associated with increased mortality and risk of recurrence. While a greater number of lymph nodes dissected was independently associated with significantly improved overall survival, the number of positive lymph nodes was independently associated with significantly worse overall survival and a trend (*p* = 0.058) towards worse recurrence-free survival. Conclusions: This study demonstrated that adjuvant CRT was independently associated with a significantly improved survival and lower risk of recurrence than observation in esophageal squamous cell carcinoma patients staged as ypT3 and/or ypN+ after receiving neoadjuvant chemoradiotherapy and radical surgery. The results of this study have implications for the design of future clinical trials and may improve treatment outcomes of patients in this setting who cannot afford or are without access to adjuvant nivolumab.

## 1. Introduction

Esophageal cancer is the seventh most commonly diagnosed cancer and the sixth leading cause of cancer death worldwide, with 604,000 new cases and 554,000 deaths in 2020 [1]. While adenocarcinoma is the most common histological type of esophageal cancer in Western countries, squamous cell carcinoma is the predominant type of esophageal cancer globally, with the highest incidence in southeastern and Central Asia [2]. Most patients with esophageal squamous cell carcinoma (ESCC) presented with locally advanced or metastatic diseases [3,4].

Neoadjuvant chemoradiotherapy followed by a planned esophagectomy, also known as trimodality therapy (TMT), is widely adopted as the standard of care for patients with resectable locally advanced esophageal cancer since the results of chemoRadiotherapy for Esophageal cancer followed by the Surgery Study (CROSS) trial prove the significant survival benefits of adding neoadjuvant chemoradiotherapy to surgery [5,6,7]. However, even treated with TMT, the prognosis of patients with locally advanced ESCC remains unsatisfactory and is distinctively poor in those who are staged as post-neoadjuvant pathologic T3/T4 (ypT3/T4; i.e., have persistence of cancer outside the esophageal wall) and/or post-neoadjuvant pathologic N+ (ypN+; i.e., failure to sterilize regional lymph node metastasis) [8]. Adjuvant therapies to improve outcomes are clearly needed; however, none has been proven effective until the publication of the results of CheckMate 577 (phase III randomized controlled trial) in April 2021, which demonstrated that adjuvant therapy with nivolumab significantly improved disease-free survival compared to the placebo (overall survival not reported) in patients with ESCC without a complete pathologic response after TMT [9]. Since then, the standard of care for these patients has shifted from surveillance to adjuvant nivolumab [10].

Nevertheless, immunotherapy is very expensive and is not covered by national health insurance or government healthcare programs in many countries, and these countries are usually the ones with a high incidence of ESCC [1,2]. In these countries, including Taiwan, most of the patients with resected ESCC after neoadjuvant chemoradiotherapy cannot afford or lack access to the recommended adjuvant nivolumab therapy. Thus, the search for other affordable, available, and effective alternative adjuvant treatments is still of great importance and interest [11].

Therefore, this study investigated whether adjuvant chemoradiotherapy (CRT) was associated with improved clinical outcomes in patients with ESCC who were staged as ypT3/4 and/or ypN+ after receiving TMT.

## 2. Materials and Methods

### 2.1. Study Participants

**Figure 1:**
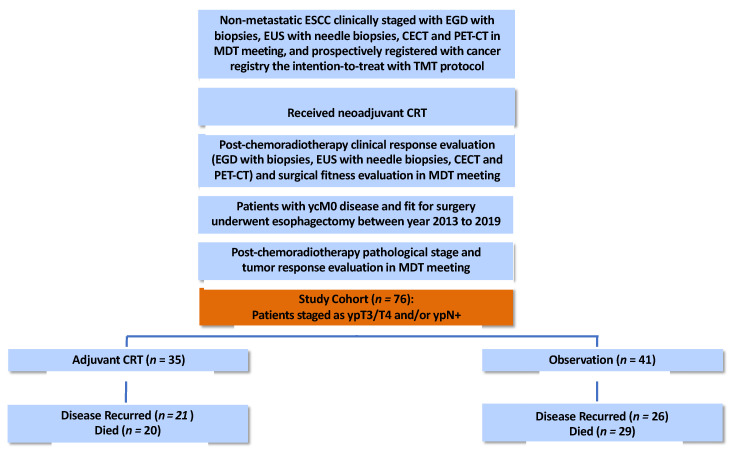
Flowchart of the patient enrollment and treatment characteristics. EGD: esophagogastroduodenoscopy, EUS: endoscopic ultrasonography, CECT: contrast-enhanced computed tomography, PET-CT: positron emission tomography-computed tomography, MDT: multidisciplinary team, TMT: trimodality therapy (i.e., neoadjuvant CRT followed by planned esophagectomy), CRT: chemoradiotherapy.

The study was approved by the Institutional Review Board (or Ethics Committee) of Chang Gung Memorial Hospital (protocol code 201900883B0 approved on 14 June 2019). This retrospective study included patients from the prospectively assembled cohort in the cancer registry of our institution. Patients were eligible for inclusion in the study if they were pathologically diagnosed with ESCC and clinically staged as non-metastatic (cM0) locally advanced ESCC, and prospectively registered in the cancer registry with the intention to be treated with neoadjuvant chemoradiotherapy (nCRT) followed by planned esophagectomy, and underwent comprehensive treatment response and surgical evaluations after nCRT, and had no interval metastasis after nCRT (ycM0), were staged as ypT3/T4 and/or ypN+ diseases after surgery between 2013 and 2019, and were deemed to be fit for adjuvant CRT in the MDT meeting (Figure 1). Patients were staged or re-staged according to the American Joint Committee on Cancer, 8th Edition. Patients were excluded if they had a history of prior or synchronous malignancy or did not receive complete pretreatment staging examinations and complete treatment response examinations according to our institutional guidelines. Both complete sets of examinations must include an esophagogastroduodenoscopy (EGD) with biopsies, an endoscopic ultrasound (EUS) with needle biopsies, contrast-enhanced computed tomography (CECT) of the neck, thorax, and abdomen, and positron emission tomography-computed tomography (PET-CT) [12,13,14].

### 2.2. Treatment Protocols

For nCRT, a total dose of 4140–5040 cGy of radiotherapy was delivered using inverse-planned static field intensity-modulated radiotherapy (sf-IMRT) or volumetric modulated arc therapy (VMAT) in the form of conventional fractionated radiotherapy (i.e., 180–200 cGy per fraction, five days per week) with concurrent chemotherapy. The chemotherapy regimens comprised six weekly cycles of intravenous carboplatin (area under curve = 2 mg/mL/min) and paclitaxel (50 mg/m^2^ body surface area) administered on the first day of each week, or two cycles of cisplatin (60–75 mg/m^2^) on day 1 combined with continuous infusion of 5-fluorouracil (800–1000 mg/m^2^) per day for 4 days starting from day 1, administered 3–4 weeks apart. The clinical target volume (CTV) in radiotherapy was delineated with a 2–5 cm longitudinal and 0.5–1.5 cm radial margins from the gross primary tumor volume (GTV-P), and a margin of 0.3–1 cm from the gross tumor volume of the metastatic lymph nodes (GTV-N). Elective nodal irradiation to the uninvolved regional lymph node area at risk for microscopic disease was highly recommended.

Treatment response evaluation and surgical evaluation were carried out 4 to 8 weeks post-nCRT in a multidisciplinary team meeting with comprehensive examinations including EGD with biopsies, EUS with needle biopsies, CECT of the neck, thorax, and abdomen, and PET/CT to exclude unfit patients from the planned subsequent surgery (e.g., patients had interval metastasis, the disease progressed to unresectable status, or was inoperable due to a decline in general condition).

The standard surgical approach consisted of a transthoracic esophagectomy with intrathoracic gastric tube reconstruction (Ivor Lewis procedure) or cervical anastomosis (McKeown procedure), a two-field lymph node dissection. A cervical lymphadenectomy was performed in selected patients who had evidence of disease in the cervical area.

Patients who had ypT3/T4 and/or ypN+ diseases after TMT were generally suggested to receive adjuvant CRT, if the patients’ postoperative conditions were deemed to be fit (i.e., patients who had serious postoperative complications that could affect the choice of adjuvant treatment were inherently excluded from this study), according to our institutional guidelines before the publication of CheckMate577 results and currently in those who cannot afford adjuvant nivolumab. Nevertheless, adjuvant CRT was offered to the patients at the discretion of the treating physician because the benefit of adjuvant CRT in this setting was not based on solid evidence and was never recommended in widely recognized international guidelines. Usually, those patients who had more additional pathologic risk factors (e.g., positive margins, a higher number of positive lymph nodes, higher Mandard Tumor Regression Grade etc.) were more likely to be strongly recommended for receiving adjuvant CRT.

For adjuvant CRT, conventional fractionated radiotherapy of 2000–3000 cGy (considering the nCRT dose and organ at risk tolerance) was delivered with concurrent chemotherapy using the same regimen previously used in nCRT. Two to four cycles of weekly carboplatin/paclitaxel regimen or one–two cycles of cisplatin/5-FU regimen were administered three–four weeks apart, and the course ended upon completion of radiotherapy. The CTV in adjuvant CRT was delineated with at least a 1 cm margin around the primary tumor bed (for ypT3/T4) and/or the involved regional nodal stations (for ypN+). Radiotherapy was delivered using inverse-planned sf-IMRT or VMAT. The attending physician determines the choice of adjuvant CRT.

### 2.3. Post-Treatment Surveillance

According to the surveillance protocol of our institution, follow-up clinic appointments were arranged every 3 months during the first 2 years, every 4–6 months during the third and fourth years, and every 6–12 months thereafter; CECT of the neck, thorax and abdomen was arranged every 3–6 months for the first 2 years, then every 6–12 months up to year 5, and EGD surveillance was arranged as clinically indicated. Clinical recurrences were defined by either biopsy or imaging (unequivocal tumor progression on subsequent exams or confirmed by two imaging modalities).

### 2.4. Statistical Analyses

All statistical analyses were performed using SPSS^®^ v. 26.0 (IBM Corp., New York, NY, USA; formerly SPSS Inc., Chicago, IL, USA). All *p*-values were two-sided, and a *p*-value of <0.05 was considered statistically significant. The median follow-up time was computed using the reverse Kaplan–Meier estimator [15]. Descriptive statistics were performed using non-parametric independent samples, median tests, chi-square tests, or Fisher exact tests, as appropriate. Propensity scores were estimated using a logistic regression model that included variables that had significantly different distributions between the unmatched groups and predictors selected a priori that were deemed to correlate with the prognoses of patients. The resulting propensity scores were then used to select controls (observation group) for matching the cases (adjuvant CRT group) in a 1:1 match fashion using the nearest neighbor approach. Survival curves for overall survival (OS, event defined as death), recurrence-free survival (RFS, event defined as any first recurrence), locoregional progression-free survival (LRPFS, an event defined as first local and/or regional recurrence), and distant metastasis-free survival (DMFS, event defined as first distant metastasis) were estimated using the Kaplan-Meier approach on the overall population and the matched pairs. As most events were expected to occur during the first two years of follow-up, the survival curves between the groups were compared using the generalized Wilcoxon test, which gives more weight to early events than later events and is thus more sensitive for detecting early differences in two survival distributions [16,17]. We also carried out univariable and multivariable Cox proportional hazard regression models to estimate the effect size of adjuvant CRT. For the multivariable model, a decision was made a priori to include the clinically relevant variables of adjuvant treatment, age, performance status, clinical stage, neoadjuvant chemotherapy regimen, neoadjuvant radiation dose, post-neoadjuvant pathologic stage, resection margin status, number of lymph nodes resected, number of positive lymph nodes, and Mandard tumor regression grade. The remaining variables were selected using backward stepwise selection with a threshold of *p* < 0.05 for entry into the model and *p* < 0.10 to stay.

## 3. Results

### 3.1. Patient and Treatment Characteristics

A total of 76 patients were identified according to the preset inclusion and exclusion criteria. The enrollment, treatment characteristics, and patient characteristics of the two groups were summarized in Figure 1 and Table 1. There was no statistically significant difference in the distribution of baseline characteristics between the two groups for age, gender, performance status, initial tumor length, tumor location, pretreatment clinical TNM-stage, neoadjuvant chemotherapy regimen, neoadjuvant RT dose, ypN classification, ypStage, resection margin status, number of lymph nodes resected, and Mandard tumor regression grade. However, significantly more patients in the adjuvant CRT group had ypT3 disease (n = 33, 88.6%) compared to the observation group (n = 23, 56.1%). In addition, 20% of the patients in the adjuvant CRT group had ypN2-3 disease in contrast to 7.3% in the observation group. The R1 resection rate was 22.9% in the adjuvant CRT group versus 9.8% in the observation group. Patients with Mandard tumor regression grade 3–4 (i.e., poor pathologic responders) comprised 74.3% of the population in the adjuvant CRT group versus 61% of the population in the observation group. The median time to adjuvant treatment after surgery was 1.6 months, and the median RT dose of adjuvant CRT was 2000 cGy, administered with a median of 3 cycles of carboplatin/paclitaxel or 2 cycles of cisplan/5-FU chemotherapy regimen.

### 3.2. Survival Analyses

The median follow-up time for the overall population was 43.4 months [95% confidence interval (CI): 29.6–57.2 months]. The median overall survival of the study cohort was 24.6 months (95% CI: 15.5–33.8 months). At the end of follow-up, 21 patients (60%) out of the 35 patients in the adjuvant CRT group had disease recurrences (locoregional and/or distant recurrence), and 20 (57.1%) of them died. In contrast, 26 patients (63.4%) out of the 41 patients in the observation group experienced disease recurrences and 29 (70.7%) of them died (Figure 1).

**Figure 2:**
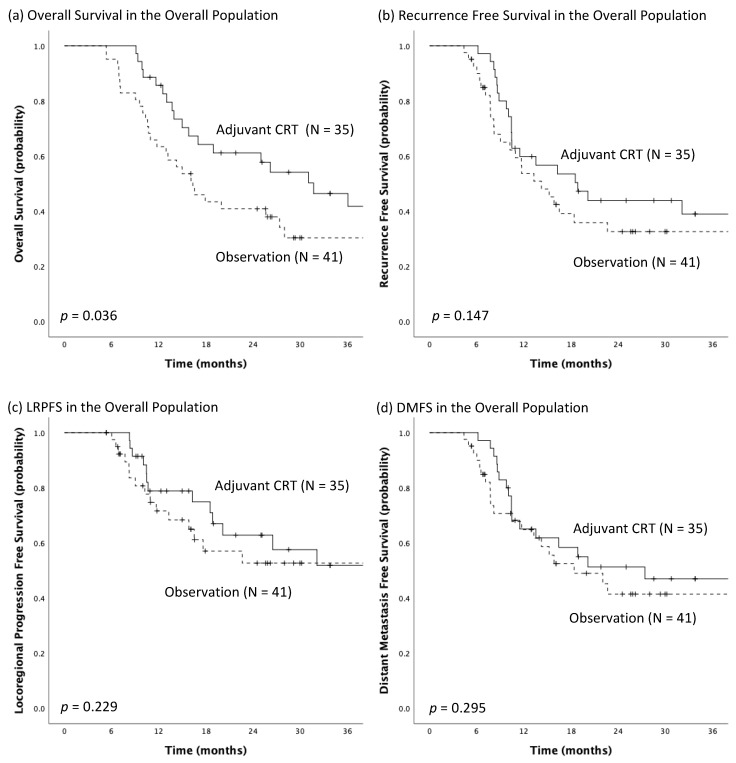
The Kaplan-Meier estimates of (**a**) overall survival, (**b**) recurrence-free survival, (**c**) locoregional progression-free survival (LRPFS) and (**d**) distant metastasis-free survival (DMFS) for the adjuvant chemoradiotherapy (CRT) group and the observation group in the overall population.

In Kaplan-Meier analyses, the median OS was 31.7 months (95% CI: 19.4–44.1 months) among patients who received adjuvant CRT and 16.3 months (95% CI: 11.9–20.7 months) among those who received observation after TMT, with the significant survival benefit toward the adjuvant CRT group most evident in the early portion (first 24 months) of the curves (*p* = 0.036; Figure 2a). The adjuvant CRT group had a median RFS of 18.9 months (95% CI: 10.2–27.6 months), while the observation group had a median RFS of 14.2 months (95% CI: 9.4–19.0 months), but the difference between the unadjusted RFS was not statistically significant (*p* = 0.147; Figure 2b). In addition, the median LRPFS of the adjuvant CRT group was not reached and the median LRPFS of the observation group was 43.4 months (95% CI: 5.0–81.8 months), yet the difference between groups was not significant (*p* = 0.229; Figure 2c). As for DMFS, there was no significant difference between groups [adjuvant CRT vs. observation, 27.4 months (95% CI: 3.7–51.1 months) vs. 18.4 months (95% CI: 9.5–27.3 months), *p* = 0.295; Figure 2d].

### 3.3. Propensity Score Matching Analyses

**Figure 3:**
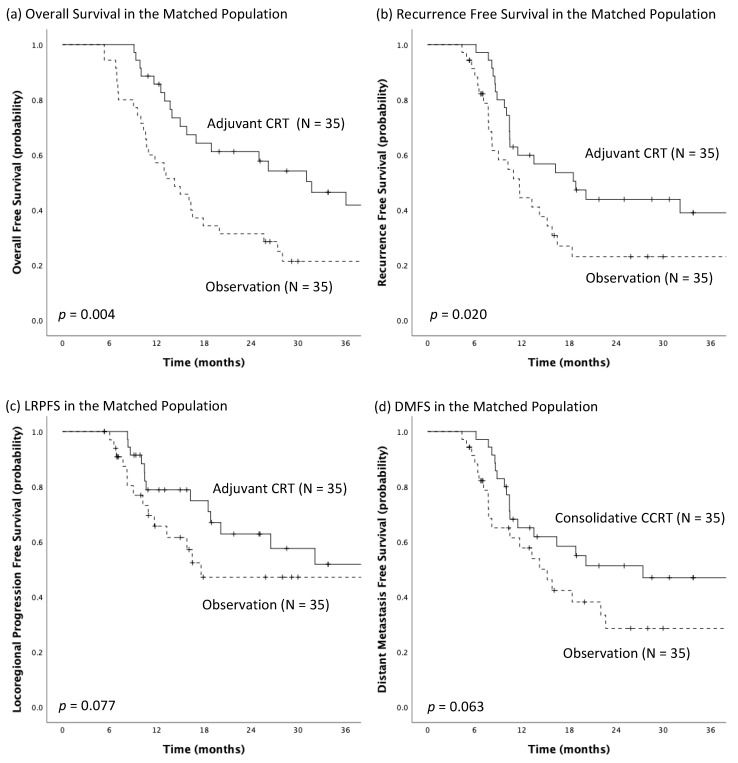
The Kaplan-Meier estimates of (**a**) overall survival, (**b**) recurrence-free survival, (**c**) locoregional progression-free survival (LRPFS), and (**d**) distant metastasis-free survival (DMFS) for the matched pairs of adjuvants chemoradiotherapy (CRT) group and the observation group.

Thirty-five matched pairs were generated by using propensity score matching for the following four pathologic prognosticators: post-adjuvant pathologic tumor classification, number of positive lymph nodes, resection margin status, and Mandard tumor regression grade. The patient characteristics in the matched population are shown in Supplementary Table S1. After propensity score matching, the distribution of these four variables became more balanced than before matching, and there was no statistically significant difference across all variables in the patient characteristics after propensity score matching. In Kaplan-Meier analysis of the matched population, the adjuvant CRT group had significantly longer median OS (31.7 months, 95% CI: 19.4–44.1 months vs. 14.3 months, 95% CI: 9.2–19.3 months; *p* = 0.004; Figure 3a) and median RFS (18.9 months, 95% CI: 4.4–27.6 months vs. 11.7 months, 95% CI: 8.1–15.2 months; *p* = 0.020; Figure 3b) than observation group. Besides, borderline significantly better LRPFS (Not reached vs. 17.6 months, 95% CI: 0.0–42.9; *p* = 0.077; Figure 3c) and DMFS (27.4 months, 95% CI: 3.7–51.1 months vs. 15.2 months, 95% CI: 10.3–20.1; *p* = 0.063; Figure 3d) in the adjuvant CRT group than in observation group were noted after propensity score matching.

### 3.4. Univariable and Multivariable Analyses

The results of the univariable analysis of the influence of the putative prognosticators selected a priori on OS and RFS were shown in Supplementary Table S2, and the results of the multivariable Cox regression model analysis were presented in Table 2. Multivariable Cox regression modeling identified adjuvant CRT as independently associated with improved OS, with a hazard ratio (HR) of 0.40 (95% CI: 0.21–0.74; *p* = 0.003), and independently associated with improved RFS, with an HR of 0.52 (95% CI: 0.28–0.95; *p* = 0.035), when adjusting the influences of other confounders in the model (Table 2). In addition, R1 resection (compared to R0 resection) and Mandard tumor regression grade 3–4 (poor responder) were also identified as independent poor prognosticators for both OS and RFS. The number of positive lymph nodes was independently associated with worse OS, while a greater number of lymph nodes dissected was independently associated with improved OS.

## 4. Discussion

Neoadjuvant chemoradiotherapy followed by planned esophagectomy is a well-established standard of care for resectable, locally advanced esophageal squamous cell carcinoma [5,6,7]. However, only about 30–50% of patients with a histological type of squamous cell carcinoma have a pathological complete response after the TMT [5,18,19]. Those who do not have a pathological complete response have a poor prognosis and it is particularly poor in those staged as ypT3/4 and/or ypN+ [8,18,19].

In this study, we only included the patients with the highest risk of death and recurrences after receiving TMT, i.e., patients staged as ypT3/4 and/or ypN+, and despite the poor prognostic factors in these patients, adjuvant CRT was independently associated with significantly longer OS and RFS, with a 60% reduction in mortality and a 48% reduction in risk of recurrence after adjusting for putative confounders. The unadjusted median survival OS was twice as long in the adjuvant CRT group as in the observation group (31.7 months vs. 16.3 months). The more favorable survival toward the adjuvant CRT group was achieved despite there being a significantly greater percentage of patients in the adjuvant CRT group than in the observation group having the pathologic poor prognosticator ypT3 (88.6% vs. 56.1%; *p* = 0.020). In addition, the adjuvant CRT group also had a non-significantly greater percentage of patients having R1 resections (22.9% vs. 9.8%), non-significantly more patients with ypN2-3 disease (20% vs. 7.3%) and non-significantly more poor pathologic responders (74.3% vs. 61%) than in the observation group. In fact, a microscopic positive (R1) resection margin and Mandard tumor regression grade 3–4 (i.e., poor pathologic responders) were independently associated with higher mortality and higher risk for recurrence in this study. In addition, after propensity score matching for the important pathologic prognosticators, the differences in the Kaplan-Meier survival curves on OS, RFS, LRPFS, and DMFS became more apparent with smaller *p*-values, and *p*-values reached a significant level for both OS and RFS. These results suggested that the association of adjuvant CRT and better survival in the patients of this study was still present after adjusting for possible confounding bias. In addition, all patients who were started on adjuvant CRT had completed the whole course without early termination of the treatment due to acute toxicity. This suggested that the low dose (2000–3000 cGy) and short course (2–3 weeks) design of the adjuvant CRT was well tolerable to patients. Besides, the post-operative clinical condition of each patient in this study was rigorously evaluated in the multidisciplinary team meeting and was suggested to be fit for adjuvant treatment. Since a low acute toxicity profile was generally expected, previous decisions on whether patients were offered adjuvant CRT was mainly based on the treating physicians’ belief in if adjuvant CRT would improve survival; and usually, those with multiple pathologic poor prognosticators were strongly recommended to receive adjuvant CRT. Even though there were non-significantly higher percentages of patients in the adjuvant CRT group who had additional poor pathologic prognostic factors mentioned above compared to the observation group, adjuvant CRT was still associated with improved survival. Our study provides physicians the crucial supporting evidence for favorable outcomes for adjuvant CRT.

A systemic review and meta-analysis examining the role of adjuvant therapy for esophageal cancer patients after neoadjuvant therapy and esophagectomy was published in January 2022 [11]. Lee et al. concluded that adjuvant therapy after neoadjuvant treatment and curative resection with negative resection margins improved OS at 1 and 5 years with moderate to high certainty of evidence [11]. However, the studies included in this meta-analysis consisted of various combinations of neoadjuvant therapies (neoadjuvant chemotherapy vs. neoadjuvant CRT) and adjuvant therapies (adjuvant chemotherapy vs. adjuvant CRT vs. adjuvant radiotherapy) [11,16,20,21,22,23,24,25,26,27,28]. Out of the 10 studies included in the meta-analysis, seven of them included patients treated with neoadjuvant CRT, and out of these seven studies, only one of them investigated the effect of adjuvant CRT [11,28]. Thus, this meta-analysis cannot answer the more specific question if adjuvant CRT can improve survival in ESCC patients who are treated with neoadjuvant CRT followed by planned esophagectomy.

Hsu et al., in a propensity score-matched analysis of 32 matched pairs of patients, reported that adjuvant chemoradiotherapy was associated with a statistically significant improvement in disease-free survival (but not statistically significant in overall survival) in pathologic non-responders (defined as patients having ypN+ or ypT classification greater than or equal to the pretreatment clinical T-classification) after neoadjuvant CRT and surgery [28]. However, the treatment timeframe of the study was between the years 2000–2012, mostly pre-dating the CROSS trial [28]. Thus, the concurrent chemotherapy regimen, mostly used in the study was Cisplatin/5-FU for both neoadjuvant and adjuvant CRT, and the radiotherapy dose for neoadjuvant CRT was 3000 cGy in 15 fractions (which is considered suboptimal dose in current practice) and for adjuvant CRT was 2340 cGy–3000 cGy in 13–15 fractions, and radiotherapy was delivered via paired anterior and posterior treatment portals or intensity-modulated radiotherapy (IMRT) [28]. In contrast, the treatment timeframe of the current study was between the years 2013–2019. More than two-thirds of the patients in the study received weekly carboplatin/paclitaxel regimen as the concurrent chemotherapy for both neoadjuvant CRT and adjuvant CRT (protocols adapted from CROSS trial), and the median dose of neoadjuvant CRT was 4500cGy in 25 fractions (which is consistent with the radiation dose of neoadjuvant CRT widely utilized in current practice), and all radiotherapy was delivered with intensity-modulated radiotherapy (mostly VMAT) to minimize radiation dose exposure to the organs at risk. To our knowledge, this is the first study to report that adding adjuvant CRT in ESCC patients who had received contemporary standard dose neoadjuvant CRT and radical surgery was associated with improved survival. 

Nonetheless, we acknowledge that this study suffers from several major limitations, including (1) the results of a retrospective study might suffer from methodological and analytical variability; (2) the small sample size of the study; and (3) selection bias and confounders could not be fully eliminated in a retrospective study. The results of this study should be validated by future prospective studies with a large sample size.

## 5. Conclusions

This study demonstrated for the first time that in patients with esophageal squamous cell carcinoma who were staged as ypT3 and/or ypN+ after receiving standard dose neoadjuvant CRT followed by planned esophagectomy, adding an adjuvant CRT was independently associated with a significantly improved survival and lower risk of recurrence than observation. The results of this study have implications for the design of future clinical trials and may improve treatment outcomes of patients in this setting who cannot afford or are without access to adjuvant nivolumab.

## Figures and Tables

**Table 1:** Patient Characteristics in the Overall Population.

Characteristics	Adjuvant CRT (n = 35)	Observation (n = 41)	*p*-Value
Age, years			0.357
Median (IQR)	51.0 (46.0–56.0)	54.0 (51.5–62.0)	
Male Sex, n (%)	35 (100%)	40 (97.6%)	1.000
ECOG Performance Status, n (%)			0.327
0	7 (20.0%)	4 (9.8%)	
1	28 (80.0%)	37 (90.2%)	
Initial Tumor Length, cm			1.000
Median (IQR)	6.0 (5.0–7.2)	5.3 (4.0–7.1)	
Tumor Location, n (%)			0.991
Upper	6 (17.1%)	7 (17.1%)	
Middle	15 (42.9%)	17 (41.5%)	
Lower	14 (40.0%)	17 (41.5%)	
Pretreatment Clinical Stage			0.200
II	1 (2.9%)	2 (4.9%)	
III	30 (85.7%)	28 (68.3%)	
IVA	4 (11.4%)	11 (26.8%)	
Neoadjuvant Chemotherapy			0.800
Carboplatin */Paclitaxel	26 (74.3%)	29 (70.7%)	
Cisplatin/5-FU	9 (25.7%)	12 (29.3%)	
Number of Cycles of Neoadjuvant Chemotherapy, Median (IQR)			
Carboplatin */Paclitaxel	6.0 (5.0–6.0)	5.0 (5.0–6.0)	0.684
Cisplatin/5-FU	2.0 (2.0–2.5)	2.0 (2.0–2.0)	1.000
Neoadjuvant RT dose, cGy			
Median (IQR)	4500 (4500–4500)	4500 (4320–4500)	0.456
ypT classification, n (%)			0.020
0	2 (5.7%)	7 (17.1%)	
1	1 (2.9%)	5 (12.2%)	
2	1 (2.9%)	6 (14.6%)	
3	31 (88.6%)	23 (56.1%)	
ypN classification, n (%)			0.391
0	16 (45.7%)	19 (46.3%)	
1	12 (34.3%)	19 (46.3%)	
2	5 (14.3%)	2 (4.9%)	
3	2 (5.7%)	1 (2.4%)	
ypStage, n (%)			0.763
II	16 (45.7%)	19 (46.3%)	
III	17 (48.6%)	21 (51.2%)	
IVA	2 (5.7%)	1 (2.4%)	
Resection margin, n (%)			0.206
R0	27 (77.1%)	37 (90.2%)	
R1	8 (22.9%)	4 (9.8%)	
Number of Lymph Nodes Resected			1.000
Median (IQR)	25 (20–33)	28 (17–35)	
Number of Positive Lymph Nodes			0.140
Median (IQR)	1 (0–2)	1 (0–1)	
Mandard Tumor Regression Grade, n (%)			0.234
1–2 (i.e., good pathologic response)	9 (25.7%)	16 (39%)	
3–4 (i.e., poor pathologic response)	26 (74.3%)	25 (61%)	
Time to Adjuvant CRT after Surgery, months			
Median (IQR)	1.6 (1.2–2.4)		
Adjuvant RT dose, cGy			
Median (IQR)	2000 (2000–2340)		
Number of Cycles of adjuvant Chemotherapy #, Median (IQR)			
Carboplatin */Paclitaxel	3.0 (2.3–4.0)		
Cisplatin/5-FU	2.0 (1.0–2.0)		

Abbreviations: CRT, chemoradiotherapy; RT, radiotherapy; 5-FU, 5-Fluorouracil; ECOG, Eastern Cooperative Oncology Group; IQR, interquartile range. * Carboplatin was replaced with Cisplatin in one patient in each group. # Adjuvant chemotherapy regimen was changed from Cisplatin/5-FU to Carboplatin/Paclitaxel in two patients and from Carboplatin/Paclitaxel to Cisplatin/5-FU in one patient.

**Table 2:** Multivariable Analysis by Cox Proportional Hazard Regression Modeling *.

		OS		RFS	
Clinical Characteristics	N	HR (95% CI)	*p*-Value	HR (95% CI)	*p*-Value
Adjuvant Treatment		0.40 (0.21, 0.74)	0.003	0.52 (0.28, 0.95)	0.035
Observation (ref)	41				
Adjuvant CRT	35				
Resection Margin		2.18 (1.04, 4.58)	0.040	2.88 (1.38, 6.02)	0.005
R0 (ref)	64				
R1	12				
Mandard Tumor Regression Grade		2.77 (1.29, 5.92)	0.009	2.22 (1.06, 4.64)	0.035
1–2 (ref)	25				
3–4	51				
No. of Positive Lymph Nodes	76	1.33 (1.14, 1.53)	0.001	1.16 (1.00, 1.34)	0.058
No. of Lymph Nodes Resected	76	0.97 (0.94, 0.99)	0.041		

* Clinically relevant variables of adjuvant treatment, age, performance status, pretreatment clinical stage, neoadjuvant chemotherapy regimen, neoadjuvant radiation dose, post-neoadjuvant pathologic stage, resection margin status, number of lymph nodes resected, number of positive lymph nodes and Mandard tumor regression grade were selected a priori and were entered into the multivariable model. The remaining variables were determined by backward stepwise selection with a threshold of *p* < 0.05 for entry into the model and *p* < 0.10 to stay. Abbreviations: HR, hazard ratio; OS, overall survival; RFS, recurrence-free survival; ref, reference group; 95% CI, 95% confidence interval; CRT, chemoradiotherapy.

## Data Availability

Data supporting reported results can be provided upon request.

## Supplementary Materials

The following supporting information can be downloaded at: https://www.mdpi.com/article/10.3390/biomedicines10112989/s1, Table S1: Patient Characteristics in the Matched Pairs. Table S2: Univariable Analysis of the Overall Population by Cox Proportional Hazard Regression.
