# Supplementary material for: Adjuvant Chemoradiotherapy Associated with Improved Overall Survival in Resected Esophageal Squamous Cell Carcinoma after Neoadjuvant Chemoradiotherapy in Intensity-Modulated Radiotherapy Era
Source: Biomedicines. 2022 Nov 21;10(11):2989. doi: 10.3390/biomedicines10112989 (PMC9687609; doi:10.3390/biomedicines10112989)
Supplement: Supplementary file 1 [file biomedicines-10-02989-s001.zip › biomedicines-1978450-supplementary.pdf]

Supplemental Table S1. Patient Characteristics in the Matched Pairs.

Characteristics	Consolidative CCRT (n = 35)	Observation (n = 35)	P-value
Age, years			.339
Median (IQR)	51.0 (46.0 – 56.0)	54.0 (52.0 – 62.0)	
Male Sex, n (%)	35 (100%)	34 (97.1%)	1.000
ECOG Performance Status, n (%)			.513
0	7 (20.0%)	4 (11.4%)	
1	28 (80.0%)	31 (88.6%)	
Initial Tumor Length, cm			1.000
Median (IQR)	6.0 (5.0-7.2)	5.4 (4.0-7.1)	
Tumor Location, n(%)			.871
Upper	6 (17.1%)	6 (17.1%)	
Middle	15 (42.9%)	13 (37.1%)	
Lower	14 (40.0%)	16 (45.7%)	
Pretreatment Clinical Stage			.198
II	1 (2.9%)	1 (2.9%)	
III	30 (85.7%)	24 (68.6%)	
IVA	4 (11.4%)	10 (28.6%)	
Neoadjuvant Chemotherapy			.792
Carboplatin*/Paclitaxel	26 (74.3%)	24 (68.6%)	
Cisplatin/5-FU	9 (25.7%)	11 (31.4%)	
Number of Cycles of Neoadjuvant Chemotherapy, Median (IQR)			
Carboplatin*/Paclitaxel	6.0 (5.0 – 6.0)	5.0 (4.3 – 6.0)	.545
Cisplatin/5-FU	2.0 (2.0 – 2.5)	2.0 (2.0 – 2.0)	1.000
Neoadjuvant RT dose, cGy			
Median (IQR)	4500 (4500 - 4500)	4500 (4140 - 4500)	.607
ypT classification, n (%)			.093
0	2 (5.7%)	5 (14.3%)	
1	1 (2.9%)	4 (11.4%)	
2	1 (2.9%)	4 (11.4%)	
3	31 (88.6%)	22 (62.9%)	
ypN classification, n (%)			.296
0	16 (45.7%)	19 (54.3%)	
1	12 (34.3%)	14 (40.0%)	
2	5 (14.3%)	2 (5.7%)	
3	2 (5.7%)	0 (0.0%)	
ypStage, n (%)			.319
II	16 (45.7%)	19 (54.3%)	
III	17 (48.6%)	16 (45.7%)	
IVA	2 (5.7%)	0 (0.0%)	
Resection margin, n (%)			.342
R0	27 (77.1%)	31 (88.6%)	

R1	8 (22.9%)	4 (11.4%)	
Number of Lymph Nodes Resected			.811
Median (IQR)	25 (20 – 33)	23 (16 - 33)	
Number of Positive Lymph Nodes			.633
Median (IQR)	1 (0 – 2)	1 (0 – 1)	
Mandard Tumor Regression Grade, n (%)			.603
1 – 2 (i.e., good pathologic response)	9 (25.7%)	12 (34.3%)	
≥ 3 (i.e., poor pathologic response)	26 (74.3%)	23 (65.7%)	
Adjuvant RT dose, cGy	Yes	No	
Median (IQR)	2000 (2000-2340)		
Number of Cycles of adjuvant Chemotherapy[#], Median (IQR)			
Carboplatin*/Paclitaxel	3.0 (2.3 – 4.0)		
Cisplatin/5-FU	2.0 (1.0 – 2.0)		

Abbreviations: CRT, chemoradiotherapy; RT, radiotherapy; 5-FU, 5-Fluorouracil; ECOG, Eastern Cooperative Oncology Group; IQR, interquartile range. *Carboplatin was replaced with Cisplatin in one patient each group.
[#]Adjuvant chemotherapy regimen was from Cisplatin/5-FU to Carboplatin/Paclitaxel in two patients and from Carboplatin/Paclitaxel to Cisplatin/5-FU in one patient.

Supplemental Table S2. Univariable Analysis of the Overall Population by Cox Proportional Hazard Regression

		OS		RFS	
Clinical Characteristics	N	HR (95% CI)	P-value	HR (95% CI)	P-value
Age, years	76	1.03 (1.00, 1.06)	.038	1.00 (0.96, 1.03)	.750
ECOG Performance Status					
0 (ref)	11				
1	65	0.71 (0.34, 1.48)	.362	0.79 (0.37, 1.70)	.5439
Tumor Location			.415		.737
Upper Third (ref)	13				
Middle Third	32	1.67 (0.71, 3.94)	.243	1.14 (0.51, 2.52)	.756
Lower Third	31	1.22 (0.52, 2.89)	.652	0.88 (0.39, 1.96)	.750
Pretreatment Clinical Stage					
II and III (ref)	61				
IVA	15	1.28 (0.65, 2.51)	.479	1.66 (0.85, 3.22)	.138
Neoadjuvant Chemotherapy					
Carboplatin*/Paclitaxel (ref)	55				
Cisplatin/5-FU	21	0.82 (0.43, 1.57)	.548	0.61 (0.30, 1.23)	.164

Neoadjuvant RT dose					
<4500 cGy (ref)	18				
≥4500 cGy	58	0.68 (0.36, 1.26)	.222	0.80 (0.41, 1.54)	.501
ypT Classification					
0-2 (ref)	22				
3	54	1.18 (0.61, 2.28)	.623	1.44 (0.71, 2.92)	.317
ypN Classification			.065		.867
0 (ref)	35				
1	31	0.90 (0.48, 1.71)	.753	0.90 (0.48, 1.68)	.742
2 and 3	10	2.40 (1.04, 5.53)	.040	1.17 (0.44, 3.10)	.759
ypStage			.521		.718
II (ref)	35				
III - IVA	41	1.12 (0.63, 2.00)	.712	0.95 (0.53, 1.70)	.855
Resection Margin					
R0 (ref)	64				
R1	12	2.35 (1.15, 4.83)	.019	3.06 (1.52, 6.18)	.002
No. of Lymph Nodes Resected	76	0.98 (0.95, 1.00)	.088	0.98 (0.95, 1.01)	.187
No. of Positive Lymph Nodes	76	1.16 (1.02, 1.33)	.028	1.08 (0.93, 1.26)	.331
Mandard Tumor Regression Grade					
1-2 (ref)	25				
≥3	51	2.76 (1.34, 5.70)	.006	2.38 (1.18, 4.81)	.016
Adjuvant Treatment					
Observation (ref)	41				
Consolidative CCRT	35	0.58 (0.32, 1.02)	.060	0.66 (0.37, 1.18)	.160

Abbreviations: HR, hazard ratio; OS, overall survival; RFS, recurrence free survival; ref, reference group; 95% CI, 95% confidence interval; RT, radiotherapy; CCRT, concurrent chemoradiotherapy.
*Carboplatin was replaced with Cisplatin in one patient in each group.
